# Translational Aspects of 3D and 4D Printing and Bioprinting

**DOI:** 10.1002/adhm.202400463

**Published:** 2024-07-09

**Authors:** Scott Taylor, Eva Mueller, Lamont R. Jones, Ashley V. Makela, Nureddin Ashammakhi

**Affiliations:** ^1^ Poly‐Med, Inc. Anderson SC 29625 USA; ^2^ Ricoh 3D for Healthcare Ricoh USA Winston‐Salem NC 27101 USA; ^3^ Department of Otolaryngology Henry Ford Heath Detroit MI 48322 USA; ^4^ Institute for Quantitative Health Science & Engineering and Department of Engineering College of Human Medicine Michigan State University East Lansing MI 48824 USA; ^5^ College of Human Medicine Michigan State University East Lansing MI 48824 USA

**Keywords:** 3D printing, 4D printing, regulatory approval, scale‐up, translational research

## Abstract

Three‐dimensional (3D) printed medical devices include orthopedic and craniofacial implants, surgical tools, and external prosthetics that have been directly used in patients. While the advances of additive manufacturing techniques in the production of medical devices have been on the rise, clinical translation of living cellular constructs face significant limitations in terms of regulatory affairs, process technology, and materials development. In this perspective, the current status‐quo of 3D and four‐dimensional (4D) (bio)printing is summarized, current advancements are discussed and the challenges that need to be addressed for improved industrial translation and clinical applications of bioprinting are highlighted. It is focused on a multidisciplinary approach in discussing the key translational considerations, from the perspective of industry, regulatory bodies, funding strategies, and future directions.

## Graphical abstract

Translational 3D and 4D printing/bioprinting involves issues related to materials development, process technology, and regulatory affairs.

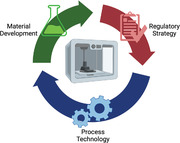

## 1 Introduction

With an aging population, rates of tissue defects and organ failure have been increasing.^[^ ^1^, ^2^ ^]^ Paralleled with shortages in donor tissues and organs available for transplantation, and the evident risk for rejection of donor tissue, the problem is magnified.^[^ ^3^, ^4^ ^]^ Because of these reasons, as well as the poor health of these patients, many do not survive the long waiting times for a transplant.^[^ ^5^ ^]^ The development of tissue engineering and regenerative medicine strategies within the last three decades have enabled us to develop products that make the replacement of several tissues possible.^[^ ^6^, ^7^ ^]^ With the exception of skin, engineering tissues and organs is still challenging, largely due to the complexity of the native tissue or organ, vascularization, size, and location within the body, to name a few.^[^ ^8^ ^]^ Although many engineered constructs may not be functional in the body, they have found use as ex vivo systems to study physiology, pathology, or test drugs and chemicals.^[^ ^9^, ^10^ ^]^ In addition, these constructs also fill gaps for studying diseases that do not have appropriate models. There have been several problems associated with engineered tissues prepared using conventional cell‐seeding approaches,^[^ ^11^ ^]^ including but not limited to nonuniform distributions within the scaffold, low cell densities, and unrealistic microenvironments for the cells. With the advent of microphysiological systems (MPSs), it became possible to develop biomimetic systems that can better recapitulate the in vivo microenvironment and build tissues in a more controllable way.^[^ ^12^, ^13^ ^]^ Among these is the adaptation of three‐dimensional (3D) printing (3DP) to 3D bioprinting (3DBP), which enabled the use of multiple biomaterials, precise control over their location, as well as cell and additive localization.^[^ ^14^, ^15^ ^]^ Additionally, the use of smart materials allows for the development of four‐dimensional (4D) constructs that can actively respond to changes in the local environment and to external and internal stimuli, making the creation of dynamic constructs possible.^[^ ^16^ ^]^ Despite advances made in bioprinting research, clinical translation has thus far been limited.^[^ ^17^ ^]^ In these nascent stages of 3D and 4D bioprinting, a lucid evaluation of the current landscape from a multidisciplinary perspective (since this is a multidisciplinary problem) supports advancement toward industrial translation and clinical application of bioprinting. Therefore, a series of special sessions in leading conferences have been organized^[^ ^18^ ^]^ to raise awareness, identify challenges, create new ideas, foster interdisciplinary collaboration, and suggest future directions to guide effective translation of important concepts and technologies for the benefit of patients on a global scale.

## 2 Mapping the Field

### 2.1 Definitions

#### 2.1.1 Microphysiological Systems

Microphysiological systems, or MPSs, include the use of small constructs, such as organoids, organ‐on‐a‐chip, engineered tissues (using conventional or 3DBP methods) and combinations thereof to be used as implantable or ex vivo devices for enhancing function, replacing lost tissues, studying physiology, or for testing chemicals, drugs, and therapeutics. MPSs may also be used for diagnostic and prognostic purposes in the future.^[^ ^19^, ^20^ ^]^ Micropathological systems (MPtSs) are MPSs that are used to study disease instead of normal physiology.

#### 2.1.2 3D Printing

3D printing, including additive manufacturing (AM), is the process of joining materials to make parts from 3D model data, typically in a layer upon layer method.^[^ ^21^ ^]^ This is directly opposed to subtractive manufacturing and formative manufacturing methodologies. There are numerous techniques available for 3D printing, including material extrusion, also called fused deposition modeling (FDM) or fused filament fabrication (FFF), binder jetting, powder bed fusion, and vat photopolymerization, to name a few.

#### 2.1.3 3D Bioprinting

3D BP is an automated deposition process typically utilizing computer‐aided design (CAD) and controlled by a software program. Different to the conventional plastics or metal AM, 3D BP allows for the printing of both biomaterials and biologically functional constituents, such as cells, in a single step and in a predefined spatial arrangement, essentially printing living structures layer by layer.^[^ ^14^ ^]^ This fabrication process creates functional, geometrically complex cellularized constructs and has numerous applications in tissue engineering, regenerative medicine, drug discovery, 3D cell culture, and other applications.

#### 2.1.4 4D Printing

While four‐dimensional (4D) printing uses the same techniques as described in 3D printing, utilizing computer‐programmed deposition of materials to create a 3D object or construct layer‐by‐layer, “4D” references the additional, predetermined ability of a construct or object to transform or change in structure, shape, properties, and even function, based on the application of internal and external stimuli, such as temperature, light, acoustic, or other environmental conditions, such as oxygen or carbon dioxide levels.^[^ ^22^, ^23^ ^]^ This structural change is attributed to the use of smart materials in the 3D printing process, making 4D printing unique for mimicking dynamic changes over time.

#### 2.1.5 4D Bioprinting

**Figure 1:**
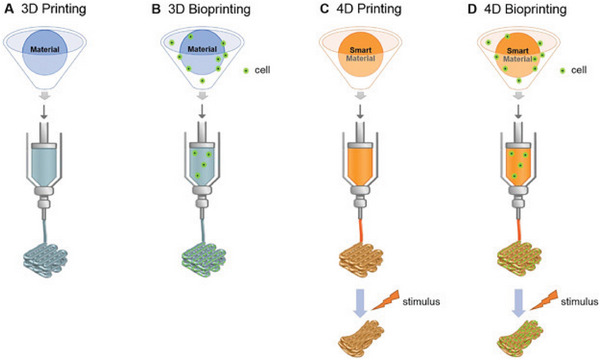
Schematic of the different printing technologies. Reproduced with permission.^[^ ^16^ ^]^ Copyright 2018, Wiley.

4D bioprinting enables the precise deposition of biomaterials and biologically active components that can undergo structural deformations over time (**Figure** **1**).^[^ ^16^ ^]^ These time‐dependent changes allow for encapsulated components to fold or unfold, and/or encapsulating and releasing drugs or cells in a programmable process. 4D bioprinting enables biologically relevant 3D constructs to mimic the dynamic changes natively found within the human body, or to control the release of encapsulated drugs for sustained drug release.

### 2.2 Current Aims of 3D and 4D Printing and Bioprinting

The current most established use of 3D printing is for surgical planning to provide a visual aid to medical professionals either before or during the surgery, or even after the surgery to assist in teaching exercises to learn about certain injuries or lesions. 3D printing medical models has become a gold standard in studying physiology and pathology of given tissues or organs and provides an excellent tool for teaching purposes. Moreover, 3D printed implants have been extensively utilized within the medical field, as a means of reinforcement and/or replacement. For example, 3D printing can be used for producing bone implants that can be custom‐made to fit a patient's specific anatomy, ensuring a perfect fit and promoting faster healing. There are several commercial and Food and Drug Administration (FDA)‐approved 3D printed bone implants available for patients and healthcare professionals, including the OsteoFab patient‐specific cranial implant (Oxford Performance Materials^[^ ^24^ ^]^), the Trabecular Titanium 3D printed implants (Stryker^[^ ^25^ ^]^), and 3D printed spinal implants (Medtronic,^[^ ^26^ ^]^ Zimmer Biomet^[^ ^27^ ^]^). There are no 4D bioprinted implants in cleared or approved medical devices at this time; however, stimulus‐responsive materials themselves are not a new concept in medical products.^[^ ^28^ ^]^

3D or 4D bioprinting is the next movement within AM to directly co‐print biological components, including cells, within a predefined model, suitable for transplantation directly into the patient. These implants can be combined with other systems, i.e., with multifluidic chips. No FDA‐approved 3D bioprinted implants exist; however, there are several companies making significant progress in the field of 3D BP. For example, Organovo focuses on creating functional human tissues for research and therapeutic applications. While they do not have FDA‐approved commercial products yet, on the pipeline includes developing bioprinted tissues for liver and kidney disease models, including support of a Phase 2 clinical trial to treat inflammatory bowel diseases, such as Crohn's Disease with a novel Farnesoid X Receptor agonist, FXR314.^[^ ^29^ ^]^ Another active company within the space of 3D BP is Aspect Biosystems. They use their 3D BP platform for the development of 3D bioprinted solutions for chronic diseases like diabetes and obesity. CELLINK is known for providing bioinks and bioprinting technologies to researchers and pharmaceutical companies, and they are actively working on developing bioprinted tissues and organs for transplantation, and their products are being used in various research projects worldwide. The current bioprinting industry is dominated by 3D Systems and BICO. CELLINK and Allevi, two companies focusing on the 3D BP technology, were recently acquired by BICO and 3D Systems, respectively. Dimension Inx received 510k FDA clearance for their CMFlex, the first 3D printed regenerative bone graft product cleared by the FDA. While there are no FDA‐approved clinical 3D bioprinted implants for direct transplantation available yet, these companies, along with many others, are making remarkable strides in the field of 3D BP.

### 2.3 Approach Scenarios

FDA‐approved 3D printers are already used in hospitals for surgical planning tools. One such example is the Stratasys J750 Digital Anatomy 3D printer which is specifically designed for medical applications, allowing surgeons to create highly realistic anatomical models for pre‐operative planning, education, and training purposes. The use of 3D printers as in situ tools, such as on‐demand skin printing, is an ongoing area of research and development. While there are no FDA‐approved 3D printers for on‐demand skin printing currently available, there have been promising advancements in the field, focusing on using 3D BP techniques to create skin grafts and other tissue constructs for wound healing and regenerative medicine applications.^[^ ^6^, ^30^ ^]^ One scenario in the future would be having bioprinters as a part of the operating room (OR) or emergency department equipment for in situ 3D BP. If patient derived cells are used with no manipulation with homologous use, FDA clearance or approval of a product may not be needed beyond that associated with the software and equipment. Although the use of unaltered patient derived cells requires no further FDA approval, there are additional challenges, in general, which should be considered. Logistics necessary to meet individual patient needs and complexity necessary to scale production can hinder opportunities for translation due to their negative impact on financial and commercial ability. Alternatively, the use of allogeneic cells which have been manipulated would require FDA approval in a similar way as for cellular engineered tissue products.^[^ ^31^ ^]^ However, allogeneic cells can be collected from a healthy donor and selected or modified, resulting in a successful transplant. For example, allogeneic iPSCs from donors with homozygous human leukocyte antigen (HLA) have been made into stock,^[^ ^32^ ^]^ allowing for HLA‐matching for recipient patients and reducing the likelihood of immune rejection.

## 3 Clinical Perspective

Primary clinical targets include implants (temporary and permanent), soft and hard tissue repair, and grafting procedures (fat graft scaffolding, extracellular matrix, tissue regeneration). Current applications are custom splints and guards, surgical guides, and planning tools, prosthetics and implants, and tissue engineering scaffolds.^[^ ^33^, ^34^, ^35^ ^]^ For example, 3D BioTherapeutics is a clinical‐stage regenerative medicine company solving medical challenges with custom‐engineered 3D bioprinted living implants.^[^ ^36^ ^]^ They began a clinical trial (now terminated by company, not safety related) to treat microtia with their 3D bioprinted ear implants.^[^ ^37^ ^]^ Another company, 3D Systems, provides surgical planning tools for patient‐matched solutions, accelerates drug development and discovery through Systemic Bio, and partners with United Therapeutics for the next‐generation bioprinted lung scaffolds.^[^ ^38^ ^]^ Medical devices produced by 3D printing include orthopedic and cranial implants, surgical instrumented dental restorations (crowns) and external prosthetics.^[^ ^39^ ^]^ While transplantation has become a significant topic of interest for clinicians, improved 3D models for pharmaceutical research and screening are equally important for improving current healthcare measures.

**Figure 2:**
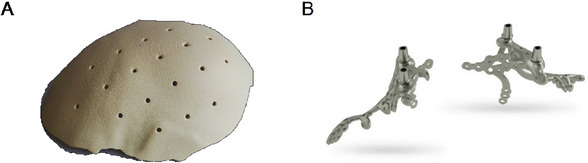
Examples of FDA‐cleared 3D printed medical devices. A) OsteoFab patient‐specific cranial medical device (Oxford Performance Materials). B) IPS Implants Preprosthetic for maxillofacial surgeries (KLS Martin). [Correction added on (16 July 2024), after first online publication: Figure 2 was updated.]

Four FDA‐approved 3D printed medical devices in hard tissue applications are highlighted in **Figure** **2** and **Table** **1**: 1) The OsteoFab cranial device (Figure 2A) is custom‐made implant produced by a 3D printer from Oxford Performance Materials (OPM). It is intended for the replacement of bony voids in the cranial skeleton for oncology and trauma cases. The design of each implant begins with obtaining patient's CT imaging data followed by CAD to determine the exact dimensions of each implant. These implants are made from OPM's proprietary OXPEKK powder formulation, and are distributed by Zimmer Biomet worldwide; 2) The Tritanium TL Curved Posterior Lumbar Cage is a 3D‐printed interbody fusion cage intended for use as an aid in lumbar fixation, produced by Stryker Corporation.^[^ ^40^, ^41^ ^]^ The cage is made of Stryker's proprietary Tritanium In‐Growth Technology, a highly porous titanium material designed for bone in‐growth and biological fixation based on rational design elements driven by pore size and structure to drive osseointegration.^[^ ^42^ ^]^ The Tritanium TL Curved Posterior Lumbar Cage is a hollow implant that consists of a unique configuration of both solid and porous structures, which are simultaneously built using AMagine, a metal powder sintering technology. The Tritanium TL Curved Posterior Lumbar Cage has a unique, curved shape and rounded teeth to facilitate multidirectional fixation. The Tritanium TL Curved Posterior Lumbar Cage received 510(k) clearance from the U.S. Food and Drug Administration in March 2018; 3) KLS Martin is a medical technology company that produces a wide range of 3D printed products, including their IPS Implants (Figure 2B) for various indications, such as cranium, orbital, mandibular, and upper extremity surgery. The IPS Implants are made from titanium powder that is fed into a laser beam and melted into the desired shape; and 4) Invisalign clear aligners are 3D printed using Align Technology and 3D System's 3D printing technology,^[^ ^43^ ^]^ used for creating a series of custom‐made aligners that are designed to gradually shift the patient's teeth into the desire position. This personalized technology realizes many advantages over traditional braces, such as improved aesthetics, comfort, and convenience.

**Table 1:** Selected medical products produced using additive manufacturing technologies, indicative examples showing trajectory in device development.

Medical device (Company)	Application	Additive technique	Raw material	FDA premarket notification reference	Year introduced
IPS implants (KLS Martin)^[^ ^44^ ^]^	Facial implants	Laser sintering	Titanium (Alloy)	510(k) K210228	2013
OsteoFab patient specific cranial device (OPM)^[^ ^45^ ^]^	Maxillofacial plating	Laser sintering	Polyether ether ketone (PEEK) Powder	510(k) K121818	2013
Osteomesh (Osteopore)^[^ ^46^ ^]^	Bone regeneration	Laser sintering	Polycaprolactone (PCL) filament	510(k) K201738	2013
Tritanium PL Cage (Stryker)^[^ ^47^ ^]^	Lumbar fusion	AMagine laser sintering	Titanium	510(k) K160955	2016
AuriNovo (3DBio)^[^ ^48^ ^]^	Auricular scaffold	Fused filament fabrication (FFF)/combination	Collagen/elastin	Clinical trial (terminated by company in 2023)	2020
Invisalign (Align Technology, 3D Systems)^[^ ^49^ ^]^	Clear aligner	Stereolithography (SLA)	Dental Clear LT resin	510(k) K981095	1999
Flux‐C (Ulrich)^[^ ^50^ ^]^	Cervical fusion	Laser sintering	Titanium	510(k) K220696	2022

## 4 Industrial Perspective

### 4.1 Current Landscape

**Figure 3:**
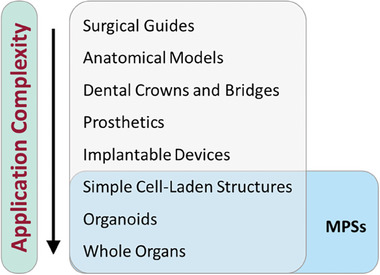
Medical targets for additive manufacturing with associated complexity.

The proliferation of AM technologies in clinically available medical devices has not been a recent phenomenon. Early medical applications (1990s — mid‐2000s), while limited in both material properties and production rate as many were formed by hand using rudimentary material preforms, identified technological potential in MPSs. This is exemplified by the successes of the Wake Forest Institute for Regenerative Medicine (with the first case of a fully printed organ, a miniature kidney in 2002)^[^ ^51^ ^]^ and from Organovo (with the first commercial bioprinter called Novogen MMX in 2009).^[^ ^52^ ^]^ While these bioprinting applications indicate the ultimate potential of AM, the majority of associated medical products rely on the design flexibility and low‐cost entry point of small batch manufacturing as justification for technology adoption.^[^ ^53^ ^]^ After expiration of key early AM patents in 2014,^[^ ^54^ ^]^ the proliferation of materials, processes, and applications allowed for rapid advancement as the cost for adoption was significantly reduced^[^ ^55^ ^]^ allowing both academic institutions and medical product companies to develop the technology toward a range of increasingly complex applications (**Figure** **3**). AM is a useful process that is more customizable compared to large‐scale industrial processes and can create highly customized, high value medical products with unique performance characteristics.

The dentistry field has embraced an additive‐first approach through the widespread adoption of digital workflows. Before the adoption of AM, dental labs already specialized in building patient specific products, for example crowns and bridges.^[^ ^56^ ^]^ In particular, dentistry labs have developed a sustainable model for producing patient‐specific implants. The proliferation of in‐office cone beam computed tomography (CBCT), inexpensive scanning equipment to digitize or directly capture oral scans, and development of dental CAD workflows has eased the transition to AM. Drill and coping guides, aligners, crowns, and bridges, for example, are typically monolithic compositions and fully dense,^[^ ^57^ ^]^ simplifying the design and manufacturing workflows. Permanent ceramic and metal products allow for autoclave sterilization.^[^ ^58^ ^]^ Tissue engineering structures as a part of MPSs are similarly segmented in terms of ready adoption of AM technology. Relatively simple, homogenous tissue structures with little vascularization or innervation, for example auricular cartilage^[^ ^15^ ^]^ and the talus bone,^[^ ^59^, ^60^ ^]^ represent the first targets for bioprinting. An example of clinical use of a targeted full bone replacement of the talus has been reported, with several dozen clinical cases reported. The talus is specifically viable because of its independence (less connection to other bones and soft tissues), responsive motion, and lesser vascularization. Longer follow‐up time points will ultimately be required to determine the relative efficacy of this approach. Organoid and tissue preforms will be in widespread clinical use before more complex or complete structures simply due to the manufacturing challenges of building multitissue, fully developed, and vascularized implants.

AM of finished medical products requires advances in three related aspects: 1) regulatory strategy, 2) process technology, and 3) materials development. AM is becoming integrated within high value industries, and a concomitant development in process technologies, from software to hardware, is moving to support this integration. With filament‐based printing, advances include multinozzle printers, blending printers, fiber‐reinforced printers, free‐form (true 3D) printing, and onboard quality inspection. Light‐based systems have advanced through the addition of multimaterial capabilities and microscale techniques such as 2‐photon polymerization. Process technology, both software and hardware, are rapidly advancing and are increasingly tailored to single processes. While proliferation of low‐cost printers lowers the investment to experiment with AM technology integration, the long‐term stability of larger organizations, whether through internal development or acquisition, leads to equipment standardization (if not homologation) and ecosystem development.

Early AM exploited readily available materials; however, as applications are increasingly tailored so does the development and adoption of materials. For example, liquid‐to‐solid AM technologies like stereolithography (SLA) require low viscosity fluids for increased precision and throughput. As AM progresses toward bioprinting, process‐compatible and bio‐compatible materials that support cellular proliferation to form tissue structures are increasingly important. Existing materials with early applications, such as polyether ether ketone (PEEK)^[^ ^61^ ^]^ and polycaprolactone (PCL),^[^ ^62^ ^]^ are limited to one or more aspects on the path to support cell‐laden 3D BP. High process temperatures associated with PEEK and PAEK AM, typically above 400 °C, are incompatible with cell‐laden and structurally sensitive materials, e.g., collagen. **Table** **2** provides example materials, categorized by material origin and novelty. In this context, material origin includes both synthetic and naturally occurring materials, wherein some naturally occurring materials that are modified for use in AM or to enhance function are included in the latter category. Novelty in this context is considered in relation to existing materials versus newly developed materials or those which have been used previously and improved upon; for example, gelatin has been used for decades in cell culture but hyaluronic acid (HA) that has been modified specifically to support AM processes is considered novel. One advantage of modified HA is that it interacts with cell surface receptors (i.e., CD44) and influences cell behavior, migration, and differentiation.

**Table 2:** Material quadrants for additive manufacturing ‐based applications.

Material	Existing	Novel	Advantages over existing
Natural	Hyaluronic acid (HA) Collagen Beta tricalcium phosphate (β‐TCP) Gelatin Alginate Cellulose Chitosan Cells, arious types	Modified HA Methacrylated collagen Silk fibroin gelatin Fibrin	Interaction with cell surface receptors Improved bioactivity, biocompatibility and biodegradability
Synthetic	Polyether ether ketone (PEEK)/ polyaryletherketone (PAEK) Metals Polyethylene Poly(L‐lactide) Polycaprolactone PLA	Photoset macromers Formlabs dental resins Polydioxanone Polyurethane Various hydrogels Polymer/ceramic composites	Bioresorbable Durability Biocompatibility Low cost

Application targets for AM technology are becoming increasingly differentiated. Early innovators, including Osteopore and Oxford Performance Materials, included AM as part of the marketing message. Clear aligners, first introduced in 1997^[^ ^63^ ^]^ and then popularized by companies including Invisalign, are prepared from intraoral scans or bite models followed by AM a positive mold, then vacuum thermoforming to create the final product. More recently, direct 3D printing of aligners is possible with advances in biocompatibility, stability, and durability of SLA resins.^[^ ^64^ ^]^ The current product pipeline is supported by advances in materials, as opposed to process technology. Recent approval of novel AM devices from a variety of materials and processes from Stratasys, Formlabs, Carbon, Ulrich, and others further adoption within the medical products industry.

At a minimum, materials for creating medical products should be appropriate for the application, and while “medical grade” does not convey a specific set of requirements, describing a material as “medical grade” or “implant grade” ideally implies the material is suited to some related purpose and supported by evidence, such as United States Pharmacopeia (USP) Class VI certification.^[^ ^65^, ^66^, ^67^ ^]^ Prior use in AM‐based or other medical products is useful, but not in itself enough to denote a material is appropriate for any given indication.

To increase successful applications and integration of AM in medical product development, several current challenges exist. Material development, integration between software and hardware, and process suitability are forefront in current research, and the impact of sterilization processes are underreported in current literature. Each aspect must be addressed to allow AM to support advanced applications for medical uses.

### 4.2 Material Challenges

The use of “medical grade” or “implant grade” terminology is often misleading. While this is an industry‐wide issue, it is a particular problem with AM technologies. With rare exception, the FDA does not clear or approve materials for medical devices, but rather an indication and the product made from these materials.^[^ ^68^ ^]^ Raw materials are prepared and controlled with the intent of medical application, and as is often the case, can conform to industry recognized USP, International Organization for Standardization (ISO), and the American Society for Testing and Materials (ASTM) standards,^[^ ^69^ ^]^ but this does not convey appropriateness for every process and application. Research in AM often repurposes existing materials that are part of existing medical product applications; however, the AM process introduces new risk factors that must be addressed, such as internal voids, phase differences, mechanics, stability, and degradation. New material developments and processability additionally require rigorous risk/benefit assessment and biocompatibility studies to determine suitability for proposed applications.^[^ ^68^ ^]^ In particular, materials that balance mechanical and functional performance with low‐temperature process windows to support bioprinting applications are an important developmental area. Smart materials for use in 4D bioprinting can solve some of these material challenges in the future due to their inherent ability to respond to internal and external stimuli by changing their physical and functional properties.^[^ ^70^ ^]^ These materials can be engineered to be highly biocompatible with specific biophysical properties, including stiffness and elasticity, to mimic the dynamic microenvironments of native tissues. The tunability also enables controlled degradation over time that can alleviate the burden upon long‐term implantation without causing serious side effects.

### 4.3 Software/Hardware Integration Challenges

Adoption from dental and orthopedic workflows have supported the first generation of AM patient‐matched implants. Workflow integration into a variety of quality, planning, and inventory (enterprise resource planning, or ERP) systems will prove an increasing and important challenge. Documentation requirements for multistep processes with handoffs between software, hardware, imaging techniques, lot traceability, and quality control analysis represent a robust engineering and efficiency challenge and are a significant driver for cost and adoption of AM‐based medical products.

New design methodologies, driven by the next generation of designers trained with additive‐native processes, will continue to drive developments and provide contrasts in capability compared to traditional manufacturing. Currently, this involves paradigms such as “lightweighting,” which are enabled through recent developments in Generative Design, artificial intelligence systems, and “black box” design automation engines. Software, for example those developed by nTop (www.ntop.com), are constraint‐driven optimization engines driven by artificial intelligence (AI) algorithms.^[^ ^71^ ^]^ Lightweighting and strength (or displacement) optimization, in particular, can reduce the amount of material implanted, and the coordination with AM allows for design element freedom while optimizing any desired outcome. With additional model training, these same models may be able to optimize designs for any number of parameters, including material selection, fluid transfer, tissue integration, and cellular functions. Generative design approaches, however, may create design validation and traceability challenges, and the technology has potential to outpace the regulatory requirements governing its use in medical applications.

### 4.4 Process Suitability Challenges

**Figure 4:**
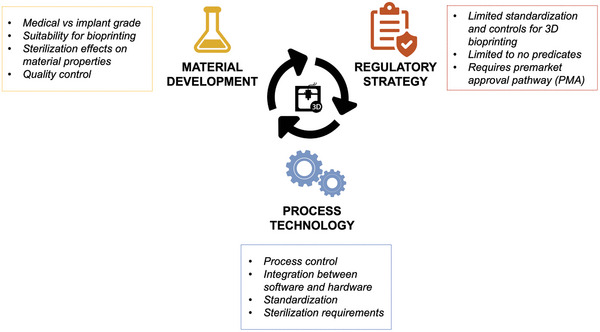
Additive manufacturing of finished medical products requires advances in three related aspects: regulatory strategy, process technology, and materials development.

Challenging implementation of AM for tissue engineering structures and bioprinting require fine control of structures and predictable performance in 3D space. Resolution is a particular challenge when creating composite cell laden MPS products. Matching process conditions to compatibility with cell‐laden constructs, primarily managing thermal load, exposure to chemical intermediates and other impurities, crosslinking conditions and reagents, and precision with creating fine structures (<0.1 mm feature size based on final application) and cell placement, is important to establishing suitable processes for advanced medical applications. Moreover, the process of which cells are introduced to bioprinting methods, sterilization, and handling prior to the AM process under controlled environmental factors are all challenges to be addressed (**Figure** **4**).

### 4.5 Sterilization Challenges

**Figure 5:**
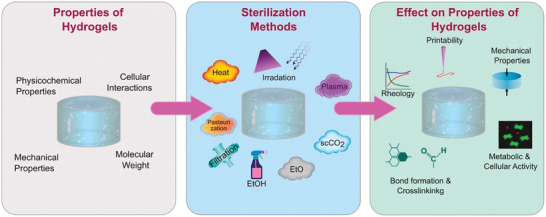
The effect of sterilization process on rheological, mechanical, physiochemical, and cytocompatibility properties on biomaterials. Reproduced with permission.^[^ ^73^ ^]^ Copyright 2023, Elsevier.

The use of AM does not preclude the use of traditional sterilization techniques (**Figure** **5**). Metal‐based products can be sterilized by autoclave and all products may potentially be sterilized through ethylene oxide (EtO) or ionizing radiation. Many of these processes, however, are expensive for small‐batch or single unit runs that often are associated with AM. In particular, plastic and composite patient‐matched implant devices, whether produced remotely or in clinic, are made on demand and are not supported by traditional industrial sterilization techniques.^[^ ^72^ ^]^ Investigators are exploring the use of sterile‐field AM through controlling the build atmosphere to minimize or eliminate the need for terminal sterilization.^[^ ^73^ ^]^ While terminal sterilization is the current gold standard for sterilizing 3D printed medical devices, more research, particularly in small batch and alternative sterilization techniques, is required to address this “last mile” problem in medical AM.

Industrial adoption of AM continues to advance medical products to support previously impossible products. These sterilization methods directly affect the properties of the hydrogels used for cell‐laden bioprinting. As shown in Figure 5, they can directly affect the printability, mechanical properties, metabolic and cellular activity, and crosslinking kinetics. It is therefore imperative to decide on the sterilization method early in the clinical translation steps to anticipate and potentially avoid these effects upon sterilization, that could eventually lead to repeat experimental work. Early, and less complex or technically challenging, applications have prepared the groundwork for composite and cell‐laden printing by developing software systems, identifying regulatory challenges, and improving printing to ultimately support applications, such as smart composites, organoid printing, organ‐on‐a‐chip for high throughput screening, and creation of other MPSs. The combination of support materials and functional biomaterials charts a path to this future, and that is where the full benefit of AM in medical products will be realized.

## 5 Funding Perspective

3D printers are used in a multitude of industries, including aerospace, medicine, and education. Both private industry and the federal government have provided funding to support these applications, from basic to applied research. With the advancements in AM, 3D BP could have applications in many biomedical fields, allowing for support through a variety of funding mechanisms including the Department of Defense (DoD), the National Institutes of Health (NIH), and the National Science Foundation (NSF), and National Aeronautics and Space Administration (NASA), to name a few. Current funding opportunities are divided between venture capital and private equity, government‐based agencies (NIH, NSF, DoD), small business innovation grant programs such as Small Business Innovation Research (SBIR) and Small Business Technology Transfer (STTR), and company‐specific grants (3D Heals). These sources provide financial support for various aspects of 3D printing research and development, such as prototyping, testing, manufacturing, clinical trials, regulatory approval, and commercialization. Some examples of current programs that offer funding are: 1) The NIH SBIR/STTR program, which supports innovative projects that address unmet medical needs or have potential to improve human health.^[^ ^74^ ^]^ Solicitations are typically addressed on the NIH website; 2) The DoD SBIR/STTR program, which supports research and development that enhance military capabilities or addresse national security challenges.^[^ ^75^ ^]^ The DoD SBIR/STTR program has funded several projects related to 3D printing medical devices and products in areas such as wound healing, bone augmentation, orthopedic implants, and surgical instruments; and 3) The National Science Foundation (NSF) SBIR/STTR program, which supports research and development that advance scientific knowledge or technological innovation.^[^ ^76^ ^]^ These three examples are merely a representation of funding opportunities available for ventures in the AM and 3D BP space in the United States (US).

## 6 Regulatory Perspective

Regulatory processes rely on the mass‐production and standardization of medical devices to ensure their compliance. However, the goal of AM is to create individualized products which are tailored to the disease as well as the patient. Not only does the technology create products which may be different each time, but they can incorporate living cells or cell‐derived products, further complicating the regulation of these living constructs. Before we can picture the translation into the clinical realm, regulatory requirements need to be satisfied, including the safety, efficacy, and clinical utility of these products.

The US FDA has Technical Considerations for Additive Manufactured Medical Devices;^[^ ^77^ ^]^ this is to serve as guidance from the FDA with regards to, broadly, manufacturing encompassing 3D printing. This document outlines technical considerations associated with the AM process, and recommendations for testing and characterization for devices that include the following key elements: design and manufacturing process considerations, environmental conditions, material controls, software workflow, and process validation. Their goal is to balance patient safety with the innovation of novel processes. Generally, drugs and medical devices are placed into specific regulatory categories, however, AM can have applications in the FDA's Center for Devices and Radiological Health (CDRH), Centre for Biologics Evaluation and Research (CBER), and Center for Drug Evaluation and Research (CDER), or a combination of these. The previous precedent for these AM products in the USA has been under the premarket notification [510(k); device found substantially equivalent to a legally marketed predicate device] and new drug application (NDA, de novo) pathways. However, now that 3D BP has become more complex, including cells and cell products (biologics), other regulatory pathways will have to be sought out including premarket approval (PMA).

Unfortunately, it is typical for the development and implementation of new regulations to come years after the first use of a new medical technology. For example, recently a clinical trial has used patient specific 3D bioprinted ears in surgical reconstruction in patients with microtia,^[^ ^48^ ^]^ the absence or misshapen ear(s) at birth. However, there is still yet to be a clear regulatory pathway for 3DBP moving forward. Although AM products could be considered under already existing regimes, they may have risk profiles different than those of other technique products. The challenges present in AM include the properties of new materials, combinations of these materials and possible inconsistencies between the production of these materials.

With AM and bioprinting becoming more advanced with applications in many biomedical fields, their use in patients will be increasing in the coming years. Many have envisioned these devices being manufactured at point‐of‐care (POC), for example, in clinical environments. This is another regulatory hurdle which will have to be addressed, with questions still surrounding risk classification, legal liabilities and FDA oversight depending on where the device is manufactured.^[^ ^78^ ^]^

## 7 Ethical Perspective

With a growing world population, the healthcare sector is facing a severe shortage of donor organs, which is expected to continue rising with the aging demographics. In the United States alone, as of 2024, over 100 000 people were awaiting solid organ transplantations.^[^ ^79^ ^]^ From a health perspective, printing more functional tissues not only allows for ex vivo or direct tissue transplantation to address the organ donor shortage but also has the capacity of improving the accuracy for in vitro drug screening, identifying both the benefits and drawbacks of new drugs sooner while decreasing the need for animal testing. The FDA recently announced that animal testing is no longer required before starting human drug trials, signaling a significant shift away from conventional animal use after more than 80 years of drugs safety regulation.^[^ ^80^ ^]^ This recent shift in testing requirements has the potential to reduce cost, address ethical concerns around getting new drugs to market and avoid potential side effects with new drugs in human clinical trials.

3D and 4D bioprinting create a unique opportunity to serve the need of reducing safety risks associated with new therapies, filling the space between traditional bench and in vitro studies and human clinical trials, and the time for clinical application. By creating MPSs, including normally functional tissue systems as well as disease specific MPtSs, screening for on‐ and off‐target responses may be performed within human‐derived and human tissue‐specific models for increased clinical relevancy. This is particularly important for drug screening to identify patient specific therapeutic response, for example in cancer therapeutic response,^[^ ^81^ ^]^ where the immediacy of clinical need and subsequent therapy selection is critical. The reduction or elimination of research animals, whether to shorten the time to personalized therapy or to reduce reliance on animals for high throughput drug discovery screening, is a significant advantage afforded by the translation of MPSs using 3D and 4D bioprinting.

## 8 Health Inequity Perspective

Translational research requires the consideration of sex and race as biological variables. However, there is often limitations in the ability to address these variables adequately during preclinical studies. MPSs can fill these gaps with the use of cells from diverse sources for bioprinting, thereby addressing inequities in health‐related research,^[^ ^82^ ^]^ broad applicability of results and their application to diverse patient populations and subsequent improved treatment outcomes. Examples include data derived from one sex and certain ethnic groups and applied to the other sex and ethnic groups, which results in inefficiency of care or even serious therapy related complications. The other example is the lack of adequate data sets for certain clinical problems that are limited to specific groups of individuals or ethnic groups. The use of MPSs will help address these disparities and will enable carrying out clinical trials on MPSs and MPtS.

## 9 Summary and Conclusions

The field of 3D printed medical devices has made significant strides in areas such as orthopedic implants, cranial implants, surgical tools, and external prosthetics for the past few decades. However, the translation of living cellular constructs using AM techniques faces various challenges. For example, the terminology of “medical grade” or “implant grade” is often misleading when we speak about materials, especially in AM technologies. While the FDA does not clear materials, but instead focuses on products and their indications, AM introduces new risk factors that require rigorous assessment and biocompatibility studies to determine suitability for proposed applications. The integration of AM in dental and orthopedic workflows has led to the creation of patient‐matched implants, but the multistep process presents challenges in quality control and efficiency. The emergence of new design methodologies, such as “lightweighting” enabled by AI‐driven optimization engines, offers potential for material reduction and design freedom, but may also pose challenges in design validation and regulatory compliance. Implementing AM for tissue engineering and bioprinting involves challenges in controlling 3D structures, managing thermal load, exposure to chemical intermediates, crosslinking conditions, and precision in creating fine structures and cell placement, as well as handling cells prior to the AM process under controlled environmental factors. By adopting a multidisciplinary approach, considering perspectives from industry, academia, regulatory bodies, funding strategies, and existing FDA‐approved medical devices, the way for improved 3D and 4D bioprinting practices and ultimately enhance patient care and outcomes will be paved. Multidisciplinary seminars and workshops at national and international conferences (for example, Society for Biomaterials) enable the improved communication between experts in different fields, all contributing toward the common goal of translating 3D/4D bioprinting into the clinic to ultimately address the growing need for organ/tissue transplantations and inclusion of gender and race differences, among many others, within translational research.

## Conflict of Interest

The authors declare no conflict of interest.
